# Characteristic of HPV Integration in the Genome and Transcriptome of Cervical Cancer Tissues

**DOI:** 10.1155/2018/6242173

**Published:** 2018-06-19

**Authors:** Weiyang Li, Yanwei Qi, Xiaofang Cui, Qing Huo, Liangxi Zhu, Aiping Zhang, Meihua Tan, Qilan Hong, Yan Yang, Huali Zhang, Chuanxin Liu, Qingsheng Kong, Jiazheng Geng, Yanjun Tian, Fancong Kong, Dongmei Man

**Affiliations:** ^1^Jining Medical University, Jining, Shandong 272067, China; ^2^Collaborative Innovation Center for Birth Defect Research and Transformation of Shandong Province, Jining Medical University, Jining, Shandong 272067, China; ^3^Affiliated Hospital of Jining Medical University, Jining, Shandong 272067, China; ^4^BGI-Shenzhen, Shenzhen 518083, China; ^5^University of Chinese Academy of Sciences, 19A Yuquan Road, Shijingshan District, Beijing 100049, China

## Abstract

High-risk HPV is clearly associated with cervical cancer. HPV integration has been confirmed to promote carcinogenesis in the previous studies. In our study, a total of 285 DNA breakpoints and 287 RNA breakpoints were collected. We analyzed the characteristic of HPV integration in the DNA and RNA samples. The results revealed that the patterns of HPV integration in RNA and DNA samples differ significantly.* FHIT*,* KLF5*, and* LINC00392* were the hotspot genes integrated by HPV in the DNA samples.* RAD51B*,* CASC8*,* CASC21*,* ERBB2*,* TP63*,* TEX41*,* RAP2B*, and* MYC* were the hotspot genes integrated by HPV in RNA samples. Breakpoints of DNA samples were significantly prone to the region of INTRON (P < 0.01, Chi-squared test), whereas in the RNA samples, the breakpoints were prone to EXON. Pathway analysis had revealed that the breakpoints of RNA samples were enriched in the pathways of transcriptional misregulation in cancer, cancer pathway, and pathway of adherens junction. Breakpoints of DNA samples were enriched in the pathway of cholinergic synapse. In summary, our data helped to gain insights into the HPV integration sites in DNA and RNA samples of cervical cancer. It had provided theoretical basis for understanding the mechanism of tumorigenesis from the perspective of HPV integration in the HPV-associated cervical cancers.

## 1. Introduction

HPV is a DNA virus that has been widely detected in humans and animals. High-risk HPV is clearly associated with cervical intraepithelial lesions and cervical cancer. Generally, about half of HPV infections could be eliminated within one year. However, infection by high-risk HPV usually could persist for several years and these types of HPV are also associated with reduced removal efficiency [1]. Moreover, persistent HPV infection for decades is likely to induce invasive cervical cancer [2].

The microscopic HPV particle is 50-60nm in diameter, and its surface consists of 72 capsomere [3]. Wrapped inside the capsid proteins is the double-stranded HPV DNA. The HPV genome may be divided into three regions, an early (E; E1, E2, E3, E4, E5, E6, E7, and E8 genes), late (L; L1 and L2 genes), and noncoding long control region (LCR). The E region is crucial for HPV replication, transcription, translation, and transformation. The L region (~2500 bp) encoded functional regulators for HPV replication and transcription [4]. Generally, absence of HPV integration in the host genome is associated with benign lesions. Positive HPV integrations are linked to cervical CIN grades and cervical cancer [5].

In recent years, high-throughput sequencing technology had provided robust means to investigate the characteristic and biological significance of HPV integration. Previous study had revealed that HPV integration could trigger genome instability; for instance, it results in genome structure rearrangement and copy number variation [6]. A recent study had shown that HPV integration within 8q24 region triggered a great number of rearrangement events in the study of HeLa cells haplotype and it might suggest HPV integration could directly initiate tumorigenesis [7]. In addition, a series of hotspots genes integrated by HPV had been found in the recent study [8]. Despite increased attention on HPV integration hotspots, the characteristic of HPV integration and the relationship between HPV integration and cervical cancer remained elusive.

In this study, a total of 285 DNA breakpoints and 287 RNA breakpoints were collected from previous studies [6, 8–12]. Our data revealed that the patterns of HPV integration in RNA and DNA samples differ significantly. Pathway analysis had revealed that breakpoints were enriched in the different pathways between RNA samples and DNA samples. Our study could further help to gain insights into the characteristic of HPV integration in DNA and RNA samples and provide theoretical basis for understanding the mechanism of tumorigenesis.

## 2. Material and Method

HPV integration sites were collected from 6 recent studies (Table S1, Table S2). Functional annotation analysis of breakpoints was performed using DAVID based on Gene Ontology and KEGG pathway databases [13, 14]. The categories of KEGG Pathways were as background databases. The breakpoints are annotated through the latest ANNOVAR in hg19 coordinates [15]. The region list of genomic elements was downloaded from the UCSC genome browser [16].

## 3. Gene Frequency

Because HPV integration was considered a strong cis-activator of flanking genes and cis-acting enhancers can influence their target genes over long distances [17, 18] (up to 1 Mb for upstream enhancers and 850 kb for downstream enhancers), breakpoints located <500 kb from annotated genes were included to calculate the affected gene frequency in HPV-integrated samples [8].

## 4. Results

### 4.1. HPV Integration Hotspots in DNA and RNA Samples

**Figure 1:**
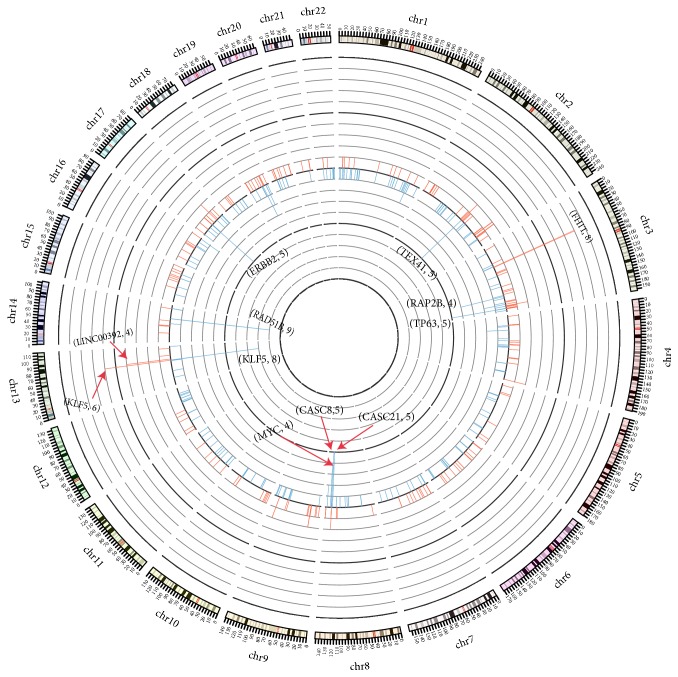
Gene frequency integrated by HPV between RNA and DNA samples. The figure showed the difference of gene frequency in DNA and RNA samples. The inner circle revealed the gene frequency (blue color) in RNA samples and the outer circle revealed the gene frequency (red color) in DNA samples. The height represented the frequency of gene integrated by HPV.

Based on frequency analysis, HPV integration hotspots had been identified in these samples.* FHIT*(8),* KLF5*(6), and* LINC00392*(4) were the most integrated genes in the DNA samples. In contrast,* RAD51B*(9),* CASC8*(5),* CASC21*(5),* ERBB2*(5),* TP63*(5),* TEX41*(5),* RAP2B*(4), and* MYC*(4) were the most integrated genes in RNA samples (Figures 1 and 4). Totally, we obtained 12 and 18 recurrent genes (frequency *⩾* 2) integrated by HPV in the DNA and RNA samples, respectively (Table S3, Table S4).

### 4.2. Distribution of Genetic Elements

**Figure 2:**
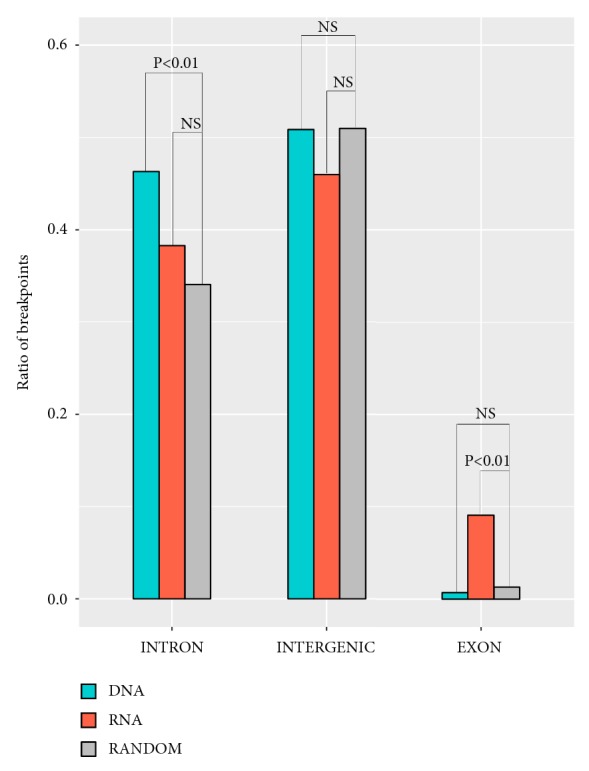
Distribution of breakpoints in genetic elements. The ratio of breakpoints in each genome element was counted. The expected ratio of each genome elements was calculated according to the random distribution of breakpoints in the whole human genome. Grey bar represented the expected ratio of breakpoints. Orange bar represented the observed ratio of breakpoints in RNA samples. Green bar represented the observed ratio of breakpoints in DNA samples. P values were calculated by Chi-squared test and were corrected by Fisher exact test.

We surveyed the distribution characteristics of the HPV breakpoints in DNA and RNA samples. The results revealed that HPV breakpoints were more prone to INTRON in the DNA samples than the RNA samples (P < 0.01, Chi-squared test, Figure 2). However, HPV breakpoints were more prone to EXON in the RNA samples than the DNA samples (P < 0.01, Chi-squared test, Figure 2).

### 4.3. Genomic Element Distribution

**Figure 3:**
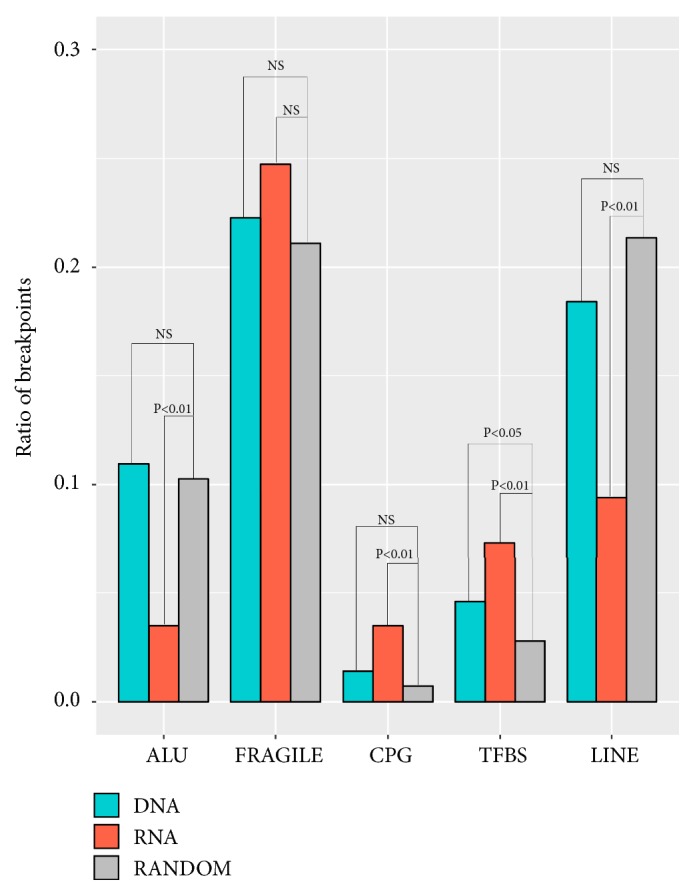
Distribution of breakpoints in the region of Fragile, CPG, TFBS, ALU, TFBS, and LINE. X axis represented different elements; Y axis represent ratio of integration breakpoints. The expected (random distribution, Grey) and the observed (actual ratio, RNA samples: orange; DNA samples: green) percentages of breakpoints are shown. P values were calculated by Chi-squared test and also were corrected by Fisher exact test.

**Figure 4:**
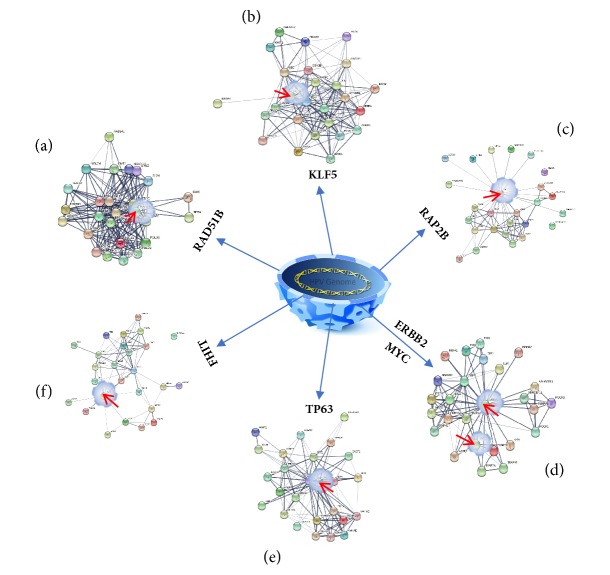
The interaction network of interacting protein for hotspot genes. The figure revealed the interaction network of hotspot genes. Line thickness indicates the strength of data support.

The HPV integration sites (breakpoints) in our RNA and DNA samples showed similar distributions in fragile, CpG, TFBS sites. However, the HPV integration sites in the RNA samples were more prone to fragile, CpG, and TFBS than that of the DNA samples (Chi-squared test, Figure 3).

### 4.4. Pathway Analysis

The results revealed that the DNA pathway was enriched on the pathway of cholinergic synapse. However, the main enriched pathways of breakpoints from RNA samples were the pathways of transcriptional misregulation in cancer, cancer pathway, and pathway of adherens junction. It revealed that there was significant difference between the enrichment pathway of RNA and DNA samples (Table S5).

## 5. Discussion

In this study, the 285 DNA breakpoints and 287 RNA breakpoints were used to carry out the bioinformatic analysis. Among 285 integration sites of DNA breakpoints, 163 integration sites were mapped by Hu and colleagues [8]. In total, Hu et al. had identified 3,667 breakpoints in 135 samples and obtained a validation rate ~83% by PCR and Sanger. However, many of the breakpoints have low integration frequencies (NNSS value < 3). Generally, the integration events with low frequencies might have fewer impacts toward tissue functions. Additionally, the breakpoints with higher NNSS values often mean more support-reads, hence greater reliability. Owing to this matter, breakpoints of NNSS value > 3 were selected in our study. In order to get the major breakpoints, we filtered out these breakpoints surrounding the major breakpoints and obtained the 163 breakpoints (Table S6).

In theory, it would be ideal to study the characteristics of HPV integration using paired DNA and RNA samples. However, there were only 5 overlapping samples between DNA and RNA samples in our study. Those small size samples were not enough to study the relation of breakpoints in the DNA and paired RNA. In addition, we had noted that it was difficult to find sufficient breakpoints (paired RNA/DNA samples) from second-generation sequencing in the existing databases. Therefore, most of the breakpoints that we used to carry out the analysis were from unpaired RNA/DNA samples.

As suggested by our results, the hotspots of HPV integration in the genome and transcriptome appeared to locate in different genes. Intriguingly, certain high frequency genes (i.e., ERBB2) in RNA samples appeared to have higher mutation frequencies (i.e., ERBB2, 5%) in the COSMIC database (http://cancer.sanger.ac.uk/cancergenome/projects/cosmic). Further, mutations in* ERBB2* had been known as therapeutic targets in lung and breast cancer in vitro [19, 20]. Our study had also observed that there were several important genes with high frequencies are preferential for HPV integration. The* RAD51B* gene belongs to the* RAD51* family, which is known to play important roles in DNA repair. Frequent HPV integrated into* RAD51B* might disrupt the DNA repair mechanism, which could partially explain the HPV-rendered genomic dysfunction and chromosome instability in cancers [21].

*FHIT* is another gene in high frequency of HPV integration, and it is located in a fragile genomic region (*FRA3B* region). This leads to a speculation that HPV integration into such region might trigger great chromosomal instability, probably via chromosomal translocation [22]. Moreover, TP63, RAP2B, KLF5, and MYC are closely related to tumorigenesis and were identified as hotspots of HPV integration [23–25]. Therefore, it is highly likely that these four genes could potentially drive tumorigenesis after genes are integrated by HPV.

As observed in the DNA samples, the HPV integration sites were inclined to INTRON region. In contrast, the HPV integration sites found in the RNA samples were enriched in EXON, CpG, and transcription factor binding sites (TFBS). Interestingly, a large portion of HPV integration sites in RNA samples was located on the no-coding region (INTRON, INTERGENIC). It might suggest that HPV integration could directly trigger the abnormal transcription and these functions of novel transcript kept unclear. Further, we found that the ratios of HPV integration sites within ALU and LINE were significantly higher in the DNA samples than those of RNA samples. Most importantly, the overall HPV breakpoints in the RNA samples suggested specific enrichments on the pathways of transcription regulation, cancer, and adherent junction. Furthermore, we noted that Xu et al. had compared the DNA junctions with the paired RNA junctions and they found that 12 of the 20 carcinomas (60%) contained a single transcriptionally active HPV16 integrate. The other 8 tumors (40%) are featured by a transcriptionally active HPV16 integrate together with one or two probably silent HPV16 integrates [12]. The phenomenon might suggest that only part of integration sites from DNA could be transcribed efficiently. The different characteristics of HPV integration in DNA and RNA might be associated with transcriptional activity of DNA breakpoints.

Due to the significant difference observed while comparing the breakpoint profiles of the DNA and RNA samples, it raises the speculations that the genomic and transcriptomic breakpoints might play the different role in tumorigenesis.

In this study, our results had revealed characteristics of HPV integration sites in the DNA and RNA samples. Additionally, the breakpoints in the RNA samples suggested tumorigenesis might arise from disrupting transcription and interrupting DNA repair mechanism. Altogether, this study had provided theoretical basis for understanding the mechanism of tumorigenesis from the perspective of HPV integration in the HPV-associated cervical cancers.

## Data Availability

The data used to support the findings of this study are available from the corresponding author upon request.

## Supplementary Materials

Supplementary 1 Table S1: the breakpoints from DNA samples.

Supplementary 2 Table S2: the breakpoints from RNA samples.

Supplementary 3 Table S3: gene frequency of breakpoints from DNA samples.

Supplementary 4 Table S4: gene frequency of breakpoints from RNA samples.

Supplementary 5 Table S5: pathway analysis of breakpoints.

Supplementary 6 Table S6: the 163 breakpoints from Hu et al.
